# Elevated Homocysteine by Levodopa Is Detrimental to Neurogenesis in Parkinsonian Model

**DOI:** 10.1371/journal.pone.0050496

**Published:** 2012-11-28

**Authors:** Jin Young Shin, Young-Hwan Ahn, Man-Jeong Paik, Hyun Jung Park, Young H. Sohn, Phil Hyu Lee

**Affiliations:** 1 Department of Neurology and Brain Research Institute, Yonsei University College of Medicine, Seoul, Korea; 2 Department of Neurosurgery, Ajou University School of Medicine, Suwon, South Korea; 3 Department of Molecular Science and Technology, Ajou University, Suwon, South Korea; 4 Severance Biomedical Science Institute, Yonsei University, Seoul, Korea; INSERM/CNRS, France

## Abstract

**Background:**

Modulation of neurogenesis that acts as an endogenous repair mechanism would have a significant impact on future therapeutic strategies for Parkinson’s disease (PD). Several studies demonstrated dopaminergic modulation of neurogenesis in the subventricular zone (SVZ) of the adult brain. Levodopa, the gold standard therapy for PD, causes an increase in homocysteine levels that induces neuronal death via N-methyl-D-aspartate (NMDA) receptor. The present study investigated whether elevated homocysteine by levodopa treatment in a parkinsonian model would modulate neurogenesis via NMDA receptor signal cascade and compared the effect of levodopa and pramipexol (PPX) on neurogenic activity.

**Methodology/Principal Findings:**

Neurogenesis was assessed in vitro using neural progenitor cells (NPCs) isolated from the SVZ and in vivo with the BrdU-injected animal model of PD using 1-methyl-4-phenyl-1,2,3,6-tetrahydropyridine. Modulation of homocysteine levels was evaluated using co-cultures of NPCs and astrocytes and PD animals. Immunochemical and Western blot analyses were used to measure neurogenesis and determine the cell death signaling. Levodopa treatment increased release of homocysteine on astrocytes culture media as well as in plasma and brain of PD animals. Increased homocysteine by levodopa led to increased apoptosis of NPCs through the NMDA receptor-dependent the extracellular signal-regulated kinase (ERK) signaling pathways. The administration of a NMDA antagonist significantly attenuated apoptotic cell death in levodopa-treated NPCs and markedly increased the number of BrdU-positive cells in the SVZ of levodopa-treated PD animals. Comparative analysis revealed that PPX treatment significantly increased the number of NPCs and BrdU-positive cells in the SVZ of PD animals compared to levodopa treatment. Our present study demonstrated that increased homocysteine by levodopa has a detrimental effect on neurogenesis through NMDA receptor-mediated ERK signaling pathway.

**Conclusions/Significance:**

Modulation of levodopa-induced elevated homocysteine by NMDA antagonist or dopamine agonist has a clinical relevance for PD treatment in terms of adult neurogenesis.

## Introduction

Recent studies have demonstrated that the adult mammalian brain has the potential to generate new neurons and to incorporate them into brain areas affected by a disease process. The neurogenesis that occurs in the subventricular zone (SVZ) and the subgranular zone of the dentate gyrus may act as an endogenous repair mechanism [1]. Several factors including neurotransmitters, growth factor, and disease states have been suggested to modulate neurogenesis [2]. Thus, modulation of endogenous neurogenesis would have a significant impact on future therapeutic strategies for neurodegenerative diseases, such as Alzheimer’s disease and Parkinson’s disease (PD). Growing evidence indicates that neurogenesis in the SVZ and hippocampus is decreased significantly in patients with PD and in animal model of PD [3]. Other findings have suggested that neurochemical deficit of dopamine and direct a-synuclein accumulation in neural precursor cells of the SVZ may influence neurogenic activity [3], [4]. The neural precursor cells in the SVZ express dopamine receptors, and several animal data suggest that dopamine-enhancing drugs increase neurogenesis in the SVZ [5], [6].

Levodopa treatment is the gold standard therapy for patients with PD, with its ability to control motor symptoms, improve quality of life, and prolong a patient’s life-expectancy. Levodopa therapy, however, causes an increase in serum homocysteine level as the drug is metabolized via catechol O-methyltransferase [7]. Hyperhomocysteinemia, the accumulation of homocysteine in the circulation, has received considerable attention in the last decade with respect to a number of disease states, since hyperhomocysteinemia is regarded as an independent risk factor for vascular diseases and cognitive impairments in elderly people [8]. Indeed, some reports have demonstrated that hyperhomocysteinemia in PD might be associated with increased prevalence of coronary artery disease, hypertrophy of carotid artery, peripheral neurodegeneration, and increased cerebrovascular resistance [9]–[12]. Although cell death mechanisms by elevated homocysteine are not clear; homocysteine- N-methyl-d-aspartate (NMDA) receptor stimulation seems to play a key role in inducing neuronal damage. Recent in vitro studies have shown that elevated homocysteine levels induced oxidative stress through NMDA receptor-mediated neuronal nitric oxide synthase activation and free radical formation, thus causing a significant increase in neuronal cell death [13], [14]. Furthermore, Rabaneda et al. [15] recently demonstrated in vitro and in vivo evidence suggesting that homocysteine inhibits the proliferation of neural progenitor cells (NPCs) in the SVZ by impairing basic fibroblast growth factor-induced proliferation. To date, no study has investigated a direct cause-effect association between levodopa-induced homocysteine elevation and neurogenic activity in the parkinsonian model. In the present study, we performed in vitro and in vivo experiments to investigate whether elevated homocysteine by levodopa would modulate neurogenesis via the NMDA receptor signaling cascade. Additionally, we performed comparative analysis of neurogenic activity between levodopa and pramipexol (PPX), a dopamine agonist.

## Materials and Methods

### Animals and Drugs Administration

All procedures of this animal research were performed in accordance with the Laboratory Animals Welfare Act, the Guide for the Care and Use of Laboratory Animals and the Guidelines and Policies for Rodent experiment provided by the IACUC (Institutional Animal Care and Use Committee) in the Yonsei University Health System. Male C57BL/6J mice (5 weeks old) were acclimated in a climate-controlled room with a constant 12 h light/dark cycle (12 h on, 12 h off) for a week prior to the initiation of drug administration. At 6 weeks of age, the mice received subchronic injections of 1-methyl-4-phenyl-1,2,3,6-tetrahydropyridine (MPTP) freshly dissolved in normal saline (25 mg/kg/day as the free base in normal saline by intraperitoneal injection (i.p).; Sigma, St. Louis, MO, USA) [16] or vehicle (normal saline, 0.1 ml/10 mg, i.p) for 5 days. On day 8 after the first MTPT injection, the mice were randomly divided into six groups (n = 4–5) and treated daily with levodopa (Sinemet®, carbidopa/levodopa, 25/100 mg. MSD, USA), PPX (1 mg, Boehringer-Ingelheim, Germany), or normal saline for 4 weeks, as follows: Group 1, control group; Group 2, normal saline-treated mice (normal saline, 0.1 ml/10 mg, i.p; MPTP-only treatment group); Group 3, levodopa-treated mice (20 mg/kg, twice daily, i.p; MPTP+levodopa treatment group) [17]; Group 4, levodopa and the noncompetitive NMDA-receptor antagonist dizocilpine (MK-801)-treated mice (10 mg/kg, once daily, ip; MPTP+levodopa and MK-801treatment group) [18]; Group 5, PPX-treated mice (1 mg/kg, twice daily, i.p; MPTP+PPX treatment group) [19]; and Group 6, PPX and MK-801-treated mice (MPTP+PPX and MK-801 treatment group). On day 28 after the first administration of levodopa, PPX, or normal saline, all animals were assigned to a BrdU injection regime following a modified protocol described by Kralic et al [20]. BrdU (Sigma, St. Louis, MO, USA) was dissolved in phosphate-buffered saline (PBS) and administered i.p once daily on five subsequent days at a concentration of 50 mg/kg. The in vivo study design is illustrated in Figure S1.

### NPCs and Astrocyte Culture

A parasagittal section was taken from the medial surface of each hemisphere of Sprague-Dawley (SD) rat embryos, and a wedge of tissue was microdissected from the portion of the lateral ventricle that included the anterior part of the SVZ [21]. The isolated NPCs were expanded in Dulbecco’s modified Eagle’s medium and Ham’s F-12 Nutrient Mixture (DMEM/F-12 mixture, 1∶1) complete media supplemented with epidermal growth factor and basic fibroblast growth factor (20 ng/mL each; both from PromoCell, Heidelberg, Germany) [22]. In the experiments, NPCs were plated on 6-well plate at a density of 1×10^4^/cm^2^ (Corning, NY, USA). Astrocytes were cultured from the cerebral cortices of 1-day-old SD rats. The dissociated cells were plated in T75 flasks. On day 14 after preparation, cells were shaken vigorously at least 3 times to detach microglia cells from astrocytes layer and removed. In the experiment, cells were plated in a transwell insert at a density of 5×10^3^/cm^2^ (Corning, NY, USA). The astrocytes were treated with varying doses of levodopa and PPX to identify a dose that did not induce cell death. For each condition, the viability of astrocytes decreased in a dose-dependent manner. Additionally, NPCs were treated with different doses of MK-801 to identify a dose that did not induce cell death (Figure S2) and SCH-23390, a dopamine 1 receptor antagonist, to identify that did not induce ERK activation (Figure S3). Accordingly, all experiments were performed using 200 µM levodopa, 1 µM PPX, 10 µM MK-801 and 50 nM SCH-23390 [23].

### Co-Cultures of NPCs and Astrocytes Treated with Levodopa or PPX

The effects of homocysteine were tested in astrocytes and NPCs that were co-cultured with no cell contact. The astrocytes treated with levodopa or PPX were maintained on a Costar transwell insert (Corning Incorporated) and the NPCs were maintained on the bottom of a 6-well plate in a humidified incubator at 37°C and 5% CO2 for 24 h or 72 h. The NPCs were then collected for assay. All experiments were replicated 3 times.

### Preparation of Plasma and Brain Samples

Blood samples were collected into EDTA-containing tubes (BD Diagnostic Systems, Sparks, MD, USA), and plasma and blood cells were separated by centrifugation (2000×g for 20 min). The isolated mouse brain and plasma were rapidly frozen and stored at −70°C until analyzed. For immunochemical analysis, all mice were deeply anesthetized with chloral hydrate (0.4 g/kg, i.p.) and then perfused with 4% paraformaldehyde in 0.1 M phosphate buffer (PB; pH 7.4). The brains were then sectioned on a sliding microtome to obtain 30-µm-thick coronal sections. All sections were stored in tissue stock solution (30% glycerol, 30% ethylene glycol, 30% 3rd D.W., 10% 0.2 M PB; pH 7.2; Sigma) at 4°C until required. For gas chromatography-mass spectrometry (GC-MS), the animals were sacrificed and the brains were rapidly frozen at −70°C.

### Measurements of Homocysteine

Hemi- brain tissues were homogenized and centrifuged (14000×g for 20 min, 4°C). The supernatant was transferred to a fresh tube for the homocysteine assay using gas chromatography-mass spectrometry (GC-MS). GC with selected ion-monitoring MS (GC-SIM-MS) runs was performed in triplicate. The mass range scanned was 50–800 U at a rate of 0.42 scan/s.

### Immunohistochemistry and Immunocytochemistry

The brain sections and co-cultured NPCs were rinsed twice in PBS and stained using specific antibodies. The primary antibodies were used as follows: mouse anti-OX-42 (1∶200 for immunocytochemistry; Serotec, Raleigh, NC, USA), mouse anti-GFAP (1∶200 for immunocytochemistry; Abcam, Cambridge, UK), rabbit anti-nestin (1∶200 for immunocytochemistry, Chemicon, Billerica, MA, USA), mouse anti-Ki67 (1∶200 for immunocytochemistry, Chemicon, Billerica, MA, USA), rabbit anti-NMDA2A receptor and NMDA2B receptor (1∶200 for immunocyto- and immunohistochemistry, Chemicon, Billerica, MA, USA), mouse anti-BrdU (1∶200 for immunohistochemistry, Roche, IN, USA). The antibodies were detected with 0.05% diaminobenzidine (DAB, Vector Laboratories, Burlingame, CA, USA) and 0.03% H2O2. Immunofluorescence labeling was carried out by incubating the cells in goat anti-mouse IgG and goat anti-rabbit IgG (both AlexaFluor-488, green and Alexa Fluor-594, red) secondary antibodies (Chemicon Chemicon, Billerica, MA, USA). The cell nuclei were counterstained with 4′,6-diamidino-2-phenylindole (DAPI; 1∶2000 dilution, Molecular Probes, Invitrogen, Carlsbad, CA, USA). The brain tissue was dried and stained with Mayer’s hematoxylin (Muto Pure Chemicals Ltd., Tokyo, Japan). The immunostained cells were analyzed using bright-field microscopy and viewed under a confocal laser scanning microscope (Olympus, Tokyo, Japan).

### Stereological Cell Counts

Unbiased stereological estimations of the total number of the stained cells in the SVZ were made using an optical fractionator, as previously described with some modifications [24]. This sampling technique is not affected by tissue volume changes and does not require reference volume determinations. The sections used for counting covered SVZ. This generally yielded 8–12 sections in a series. Sampling was performed with the Olympus C.A.S.T.-Grid system (Olympus Denmark A/S, Denmark), using an Olympus BX51 microscope, connected to the stage and feeding the computer with the distance information in the z-axis. A counting frame (55%, 53, 1281 µm^2^) was placed randomly on the first counting area and systematically moved though all counting areas until the entire delineated area was sampled. Actual counting was performed using a x100 oil objective. Guard volumes (4 µm from the top and 4–6 µm from the bottom of the section) were excluded from both surfaces to avoid the problem of a lost cap, and only the profiles that came into focus within the counting volume (with a depth of 10 µm) were counted. The total number of stained cells was calculated according to the optical fractionator formula [25].

### Total RNA Extraction and Reverse Transcriptive PCR (RT-PCR)

Total RNA was extracted from the NPCs using Trizol reagent (Invitrogen, Carlsbad, CA, USA) in accordance with the manufacturer’s protocol. An equal amount of RNA (approximately 1 µg) in each experiment was reverse transcribed using an amfiRivert cDNA Synthesis Premix (GenDEPOT, Barker, TX, USA). Subsequently, 2 µl of cDNA was used as a template for RT- PCR analysis in amfiRivert 1-Step RT-PCR Kit. (GenDEPOT, Barker, TX, USA). The PCR reaction was performed using 10 pmol each of the primers for NMDA receptor 1 (NR1; forward 5′-CTG CAA CCC TCA CTT TTG AG-3′, reverse 5′-TGC AAA AGC CAG CTG CAT CT-3′), NMAD 2A receptor (NR2A; forward 5′- GAC GGT CTT GGG ATC TTA AC-3′, reverse 5′- TGA CCA TGA ATT GGT GCA GG-3′), NMAD 2B receptor (NR2B; forward 5′- CAA GAA CAT GGC CAA CCT GT-3′, reverse 5′- GGT ACA CAT TGC TGT CCT TC-3′), NMAD 2C receptor (NR2C; forward 5′- TGG AAA CTT CGA CAC TCG GT-3′, reverse 5′- TCC AAA GAG CTG CTC ACG TC-3′) [26]. After an initial denaturation at 94°C for 5 min, 30 cycles of PCR were performed, consisting of denaturation (30 s, 94°C), annealing (1 min, 58°C [NMDA receptor 1], 55°C [NMDA receptor 2A, NMDA receptor 2B], 57°C [NMDA receptor 2C]), extension (1 min, 72°C) followed by a final extension (10 min, 72°C). The PCR products were separated by electrophoresis on 2% agarose gel and stained with ethidium bromide. Gels were examined under UV illumination.

### Flow Cytometric Measurement of Cell Death Using Annexin-V/PI

Flow cytometric analysis was performed to evaluate the extent of apoptotic and necrotic cells. Co-cultured NPCs were harvested and stained with Annexin/PI using kit (BD, San diego, CA, USA). We counted only Annexin V-positive cells in the right-lower quadrant (apoptotic cells). Samples were immediately kept on ice and analyzed on FACS. Data was acquired and analyzed using Winmdi software (http://www.cyto.purdue.edu/flowcyt/software/Winmdi.htm). Acquisition gates were wet using the forward and side light scatter of the cells and a minimum of 10,000 events were collected for each sample.

### Caspase-3 Activity Assay

Caspase-3 activity was measured by monitoring the proteolysis of corresponding colorimetric substrates using a caspase-3 activity assay kit (Chemicon, Billerica, MA, USA). Co-cultured NPCs were collected and washed in ice-cold PBS (pH 7.0) and subsequently lysed in 1× lysis buffer for 10 min in ice; the lysates were clarified by centrifugation at 16,000×g for 10 min. After centrifugation for 10 min, cytosolic extracts of NPCs were transferred to a fresh tube and put on ice. Then 30 µg of the caspase-3-specific colorimetric substrate acetyl-Asp-Glu-Val-Asp-7-p-nitroaniline (Ac-DEVD-p NA) was added to the cytosolic extracts. The extracts were incubated for 1 h at 37°C. The release of DEVD-p NA was quantified at 405 nm using an enzyme-linked immunosorbent assay (ELISA) plate reader.

### Western Blot Analysis

For western blotting, co-cultured NPC were harvested and extracted using lysis buffer containing protease inhibitor (iNtRON Biotechnology, SeongNam, S. Korea). Briefly, 50 µg of protein were separated using SDS-gel electrophoresis and transferred to nitrocellulose membrane (Amersham, Piscataway, NJ, USA). The membranes were blocked in non-fat milk. Membranes were probed with 1∶1000 dilutions of the following primary antibodies: rabbit polyclonal the extraceullar signal-regulated kinase (ERK)1/2 and rabbit polyclonal phospho-ERK1/2 (Cell Signaling, Danvers, MA, USA). As a secondary antibody, a 1∶2000 dilution of horseradish peroxidase-conjugated goat anti-rabbit antibody (Zymed Laboratories, San Francisco, CA, USA) was used. Antigen-antibody complexes were visualized with a chemiluminescence system (Amersham, Piscataway, NJ, USA), followed by exposure to X-ray film (Kodak X-OMAT, Rochester, NY, USA). For semiquantitative analysis, the densities of the immunoblot bands were measured by computer imaging (Image J; NIH, Bethesda, MD, USA). All proteins were normalized with actin.

### Statistical Analysis

The group means were compared using the Mann-Whitney U-test for pairs and the Kruskal-Wallis analysis for multiple groups. P-values less than 0.05 were considered statistically significant. Statistical analyses were performed using commercially available software (version 12.0; SPSS Inc., Chicago, IL).

## Results

### Phenotype and Proliferative Capacity of the Cultured NPCs

**Figure 1:**
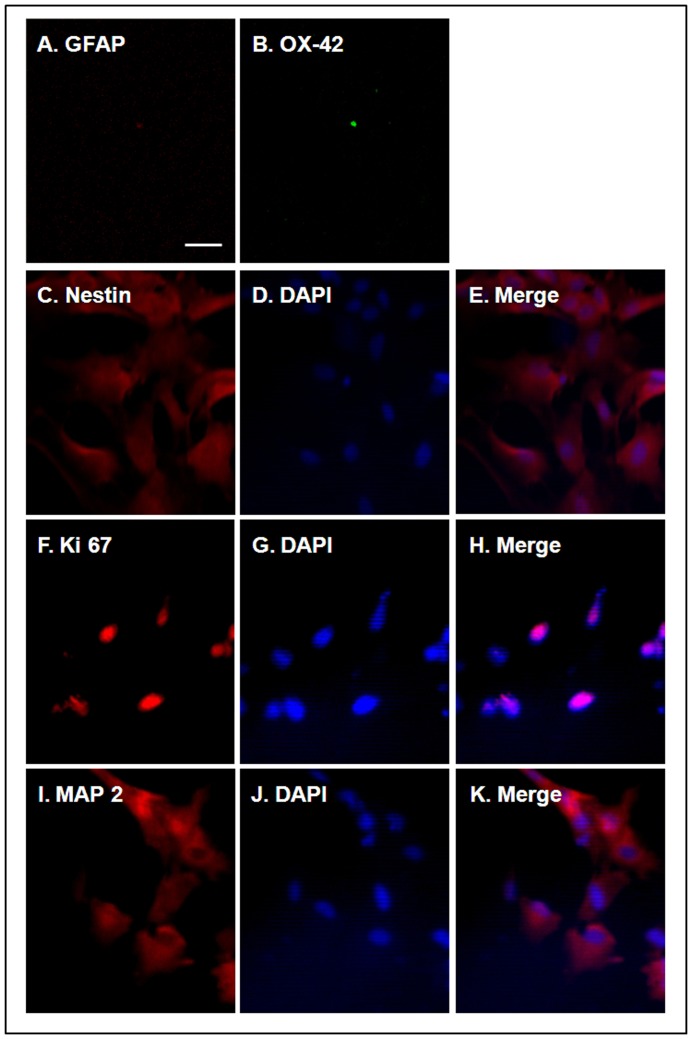
Phenotype and proliferative capacity of cultured NPCs. NPCs isolated from the SVZ exhibited no GFAP-positive or OX-42-positive cells (A and B). Most NPCs were immunostained with nestin (C and E), Ki67 (F and G), and MAP 2 (I and K). These marker-positive NPCs were visualized using fluorescent secondary antibodies conjugated to DAPI (D, G, and J). Scale bar: 10 µm.

The phenotypic properties of the NPCs were determined using nestin, the neural stem cell marker, to reveal cells expressing a neuronal phenotype, whereas GFAP (an astrocyte marker) and OX-42 (a microglia marker) were applied to reveal the presence of glial cells. No GFAP-positive or OX-42-positive cells were observed in the cultured NPCs (Fig. 1A and B), and most of the NPCs were nestin-positive (Fig. 1C). Additionally, the NPCs were immunostained with Ki67 (Fig 1F, G, H), a proliferation marker and MAP 2 (Fig 1I, J, K), a neuronal marker.

### mRNA Expression and Immunodetection of NMDA Receptor Subunits in Cultured NPCs

**Figure 2:**
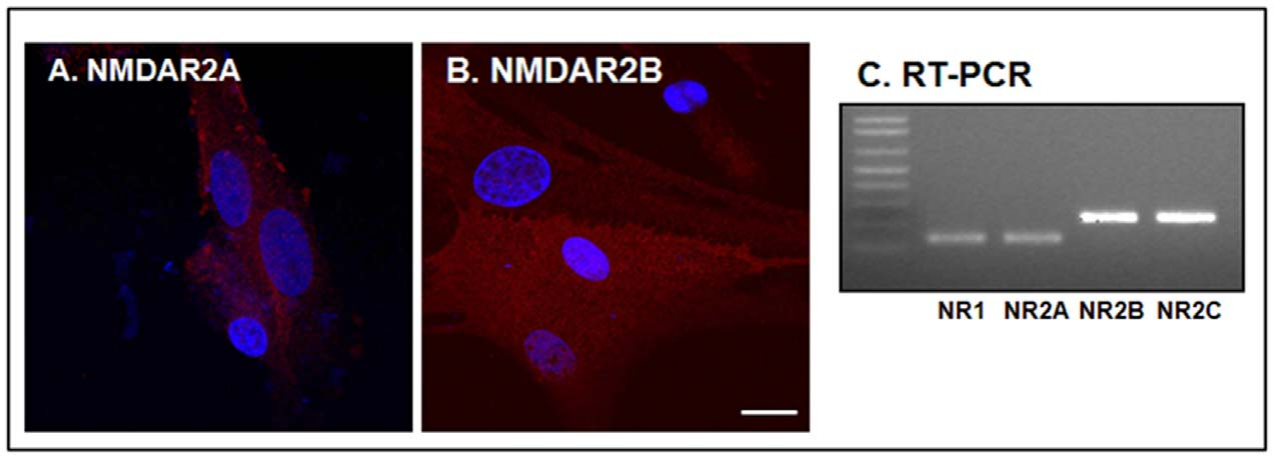
Expression of NMDA receptor subunits in NPCs. Immunocytochemistry revealed that the NPCs expressed NMDA receptor 2A and 2B subunits (A and B). RT-PCR showed that NPCs expressed NMDA receptor 1, 2A, 2B, and C subunits (C). Scale bar: 20 µm.

Immunocytochemistry revealed that NPCs expressed NMDA receptor NR2A and NR2B subunits (Fig. 2A and B). These results were confirmed by RT-PCR showing total RNA isolated from NPCs expressing detectable NR1, NR2A, NR2B, and NR2C (Fig. 2C).

### Levodopa Treatment Leads to Increased Release of Homocysteine on Astrocyte Culture Media

**Figure 3:**
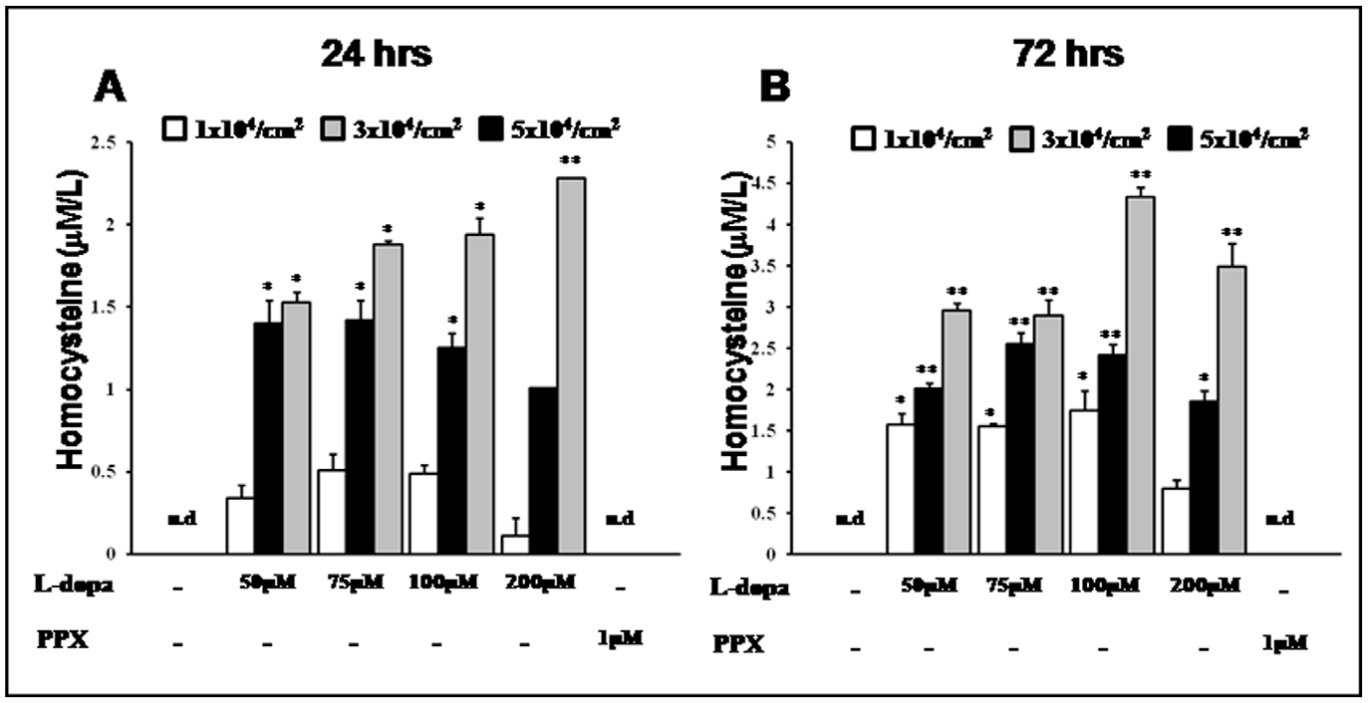
Levodopa-induced release of homocysteine from astrocytes. Levodopa treatment led to increase in extracellular concentration of homocysteine in a time-dependent manner (A and B). Release of homocysteine by levodopa treatment was also dependent on the number of astrocytes with the maximum level in the highest dose of levodopa. Homocysteine was not detectable in the astrocyte culture media after PPX treatment (A and B). Homocysteine was detected by GC-mass. Values are means ± SD (n = 4/group, *P<0.005).

The astrocyte culture media was treated with levodopa to determine its effect on homocysteine levels. The extracellular concentration of homocysteine increased linearly in a time-dependent manner during incubation with levodopa and reached its highest level at 72 h after levodopa treatment. Levodopa-induced homocysteine release was dependent on the number of astrocytes with the maximum level in the highest dose of levodopa (Fig. 3A and B). Homocysteine was not detectable in astrocyte culture media after PPX treatment (Fig. 3A and B) and in levodopa-treated NPC culture media without astrocyte (data not shown).

### Levodopa Treatment Induces Apoptotic Cell Death in NPCs

**Figure 4:**
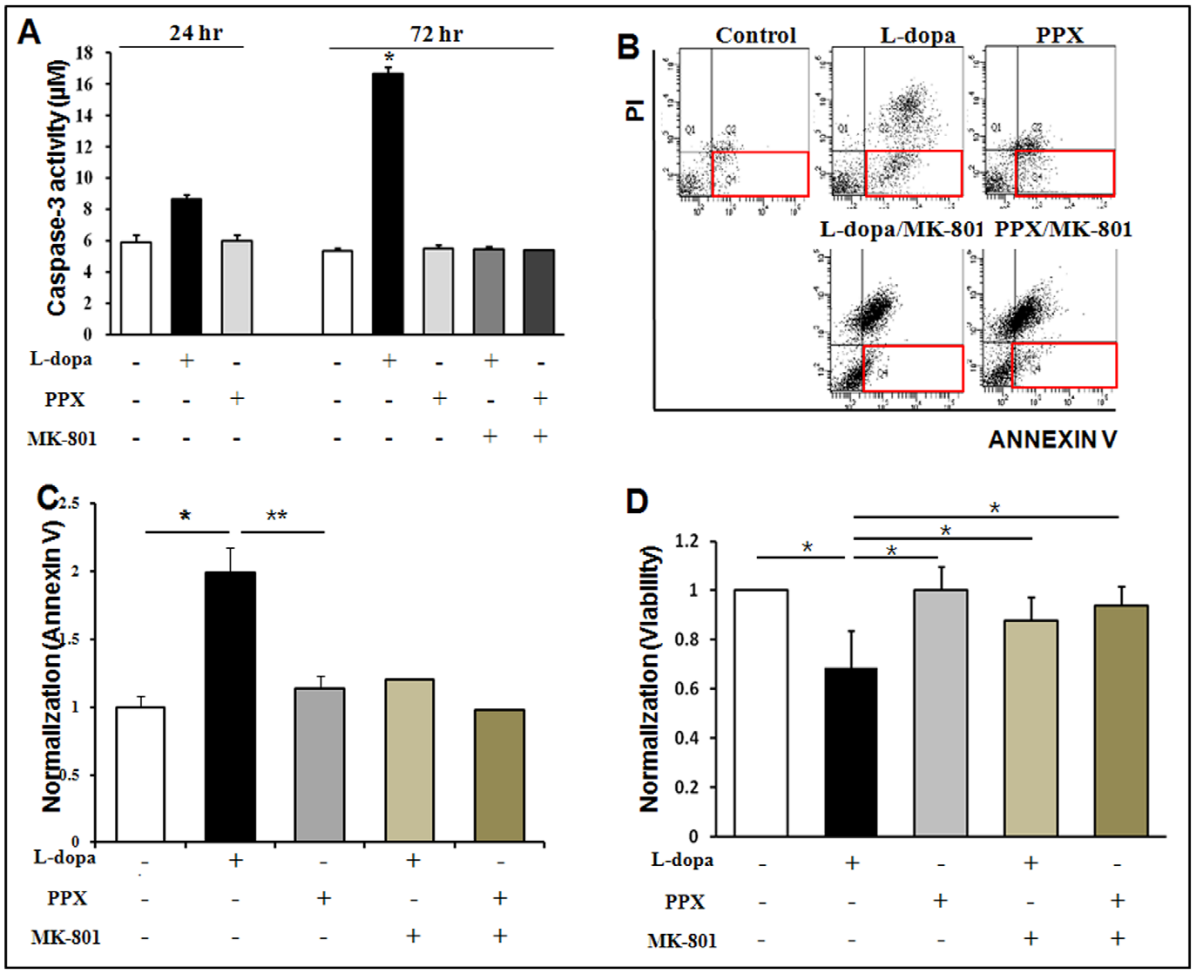
Levodopa treatment induced apoptotic cell death in NPCs. Levodopa treatment significantly increased caspase-3 activity in a time-dependent manner in NPCs. Moreover, caspase-3 activity was significantly higher in the levodopa-treated NPCs than in the control or PPX-treated NPCs at 72 h (A). The flow cytometric assays revealed that Annexin-V- positive cells increased significantly in the levodopa-treated NPCs compared to the control and PPX-treated NPCs (B). The quantitative analysis revealed a significant increase in apoptotic cell death in the levodopa-treated NPCs compared to the control or PPX-treated NPCs (C). NPC viability decreased significantly in levodopa-treated NPCs compared to that in the control and PPX-treated NPCs (D). However, administration of MK-801 to the levodopa-treated NPCs significantly decreased caspase-3 activity, Annexin-V-positive cells, and apoptotic cell death compared to those in the levodopa-only NPCs. On the other hand, MK-801 had no significant effect on apoptotic cell death in PPX-treated NPCs (B-D). Values are means ± SD (n = 4/group, *P<0.005).

The NPCs were treated with different doses of homocysteine to evaluate the direct effects of homocysteine on the viability of NPCs measured by trypan blue cell counting. Homocysteine treatment decreased the viability of NPCs in a dose-dependent manner (Figure S3 A). Next, NPCs were co-cultured with levodopa- or PPX-treated astrocytes for 24 h and 72 h, respectively, to determine whether levodopa stimulated an increase in homocysteine-activated apoptosis. Caspase-3 activity significantly increased in a time-dependent manner in levodopa-treated NPCs and was significantly higher than that in the control or PPX-treated NPCs at 72 h (Fig.4A). Furthermore, the flow cytometric assay using Annexin V/PI revealed that Annexin-V-positive cells, a cluster in the right-lower quadrant, had significantly increased in the levodopa-treated NPCs compared to the control and PPX-treated NPCs (Fig. 4B). A quantitative analysis performed using the flow cytograms revealed a significant increase in apoptotic cell death in the levodopa-treated NPCs compared to that in the control and PPX-treated NPCs (Fig. 4C). NPC viability had significantly decreased in the levodopa-treated cells as compared to that in the control and PPX-treated cells (Fig. 4D). MK-801, a NMDA antagonist, significantly decreased caspase-3 activity, the number of Annexin-V-positive cells, and cell death in levodopa-treated NPCs (Fig. 4B, C, D), whereas MK-801 had no significant effect on apoptotic cell death in PPX-treated NPCs (Fig. 4B, C, D).

### Effects of Levodopa Treatment on the Regulation of ERK Signaling

**Figure 5:**
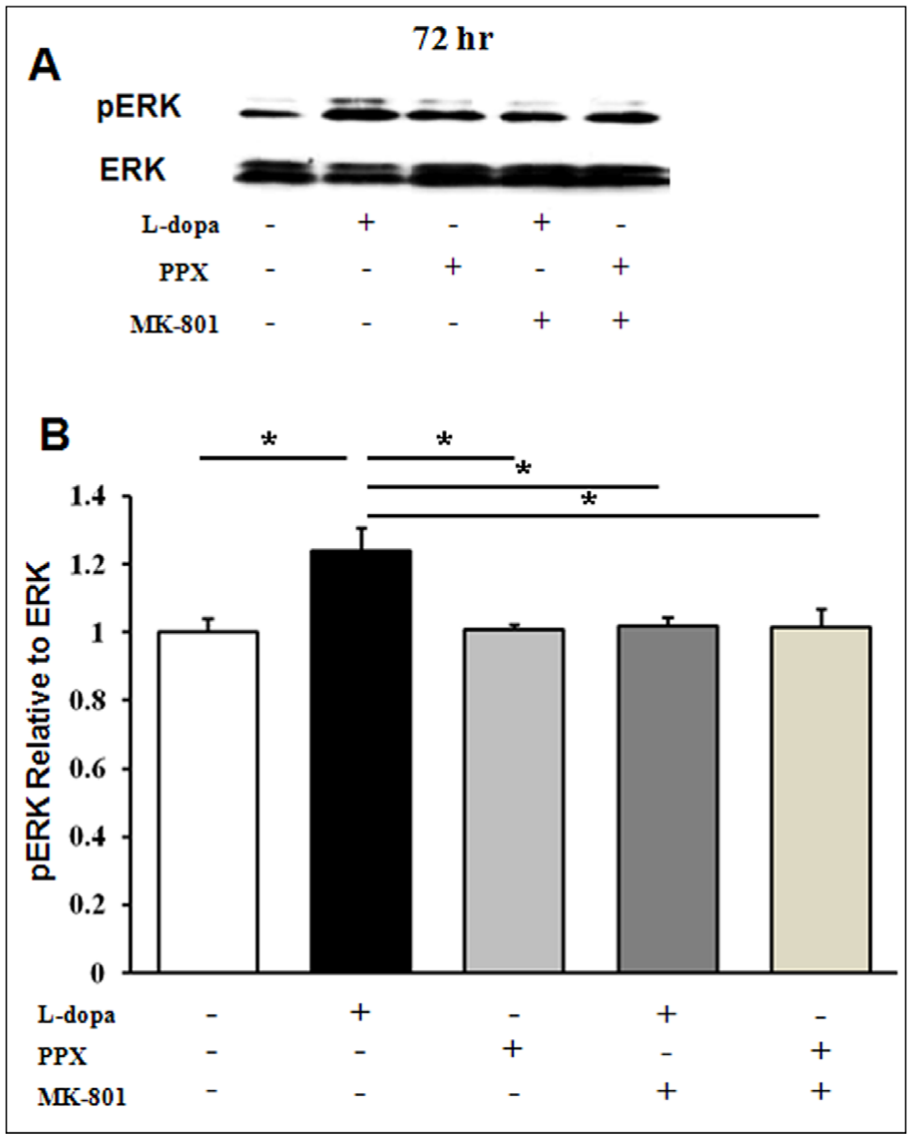
Effects of levodopa treatment on the regulation of ERK signaling. The phosphorylated form of ERK increased significantly in levodopa-treated NPCs compared to that in the controls (A and B). MK-801 treatment in the levodopa-treated NPCs significantly attenuated the expression of phosphorylated ERK compared to that of the levodopa-only NPCs (A and B). The expression of phosphorylated ERK did not change significantly in PPX- treated NPCs compared to the controls. The results represent three replications in each group. *P<0.02.

ERK signaling was assessed by western blot analysis because homocysteine, as agonist of NMDA receptor, can lead to the phosphorylation and subsequent activation of ERK signaling in neurons. Direct treatment of homocysteine in the NPCs increased the expression of phosphorylated ERK in a dose-dependent manner but the expression of phosphorylated ERK was decreased in presence NMDA antagonist, MK-801 (Figure S3 B and C). Then, NPCs were co-cultured with levodopa or PPX-treated astrocytes for 72 h to investigate whether increased levels of homocysteine modulated regulation of ERK signaling pathways through the NMDA receptor. The phosphorylated form of ERK was significantly higher in levodopa-treated NPCs compared to controls (Fig. 5A and B). However, the expression of phosphorylated ERK was attenuated in levodopa-treated NPCs that received MK-801 treatment compared to the levodopa-only NPCs (Fig. 5A and B). To evaluate that ERK is not induced by D1 receptor simulation, the NPCs co-cultured with levodopa treated- astrocytes were treated with 50 nM D1receptor antagonist (SCH-23390) for 72 h. D1 receptor antagonist treatment did not decrease the expression of phosphorylated ERK, suggesting that ERK activation is not mediated via D1 receptor on the NPC (Fig S3 D). The expression of phosphorylated ERK did not significantly change in PPX-treated NPCs compared to the controls (Fig. 5A and B).

### Levodopa Treatment Leads to Increased Homocysteine Levels in Plasma and Brain of PD Animals

**Figure 6:**
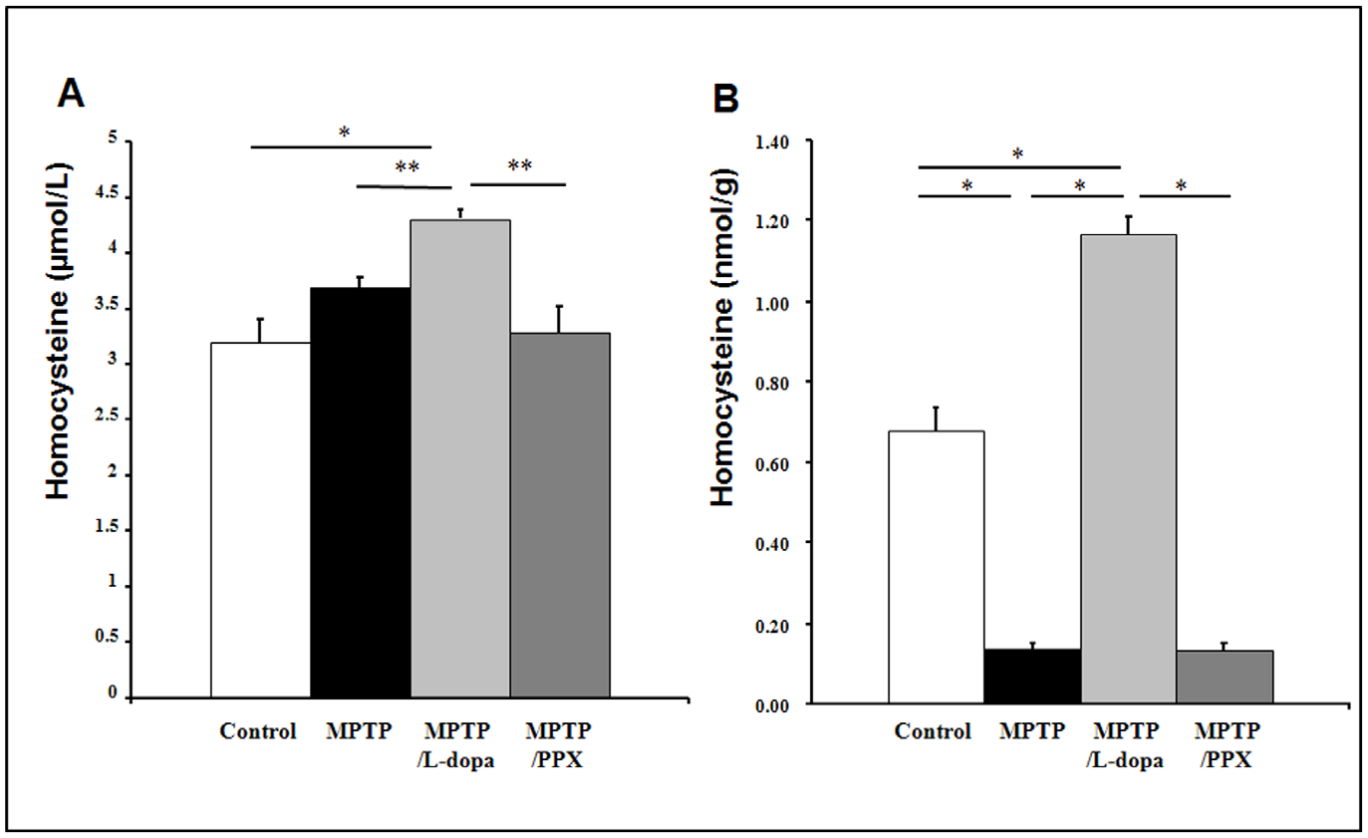
Levodopa treatment led to increased levels of homocysteine in both plasma and brain Levodopa treatment in MPTP-treated mice produced a significant increase in plasma (A) and brain (B) levels of homocysteine compared to that of the control, MPTP-only, and MPTP+PPX- treated mice (A). Values are means ± SE (n = 5; *P<0.05, **P<0.01).

Plasma homocysteine levels in MPTP-treated mice and MPTP+PPX-treated mice were not significantly different from those of the control group. However, levodopa treatment significantly increased plasma homocysteine levels in MPTP-treated mice compared to the control group, MPTP-only, and MPTP+PPX-treated mice (Fig. 6A). Moreover, levodopa administration significantly increased the levels of brain homocysteine in MPTP-treated mice compared to that of the control, MPTP-only, and MPTP+PPX-treated mice, whereas the concentration of brain homocysteine was significantly reduced in the MPTP-only and MPTP+PPX-treated mice compared to the controls (Fig. 6B). In MPTP untreated mice, levodopa treatment or co-administration of MK-801 and L-dopa in MPTP significantly increased plasma and brain homocysteine levels compared to controls (Fig. S4 A).

### Comparison of Neurogenic Activity Between Levodopa and PPX in MTPT-treated PD Animals

**Figure 7:**
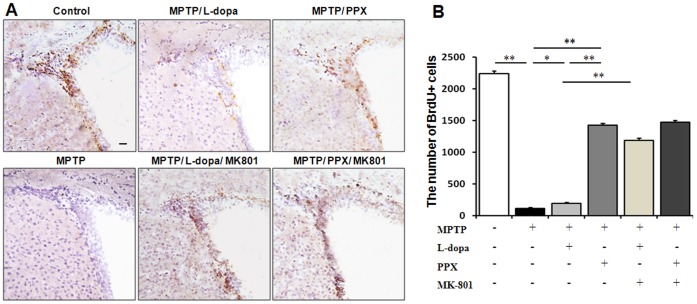
Comparison of neurogenic activity between levodopa and PPX in MTPT-treated PD animals. The immunohistochemistry analysis revealed that the number of BrdU-positive cells decreased significantly in MPTP-treated mice compared to control mice, whereas levodopa or PPX treatment increased BrdU-positive cells in MPTP-treated mice compared to MPTP-only treated mice (A). The stereological analysis revealed that the number of BrdU positive cells was much greater in the PPX-treated mice than in the levodopa-treated mice (B). MK-801 administration significantly increased the number of BrdU-positive cells in the SVZ of the levodopa-treated PD mice compared to the levodopa-only PD animals (A and B), whereas no significant changes in neurogenic activity were observed in the PPX-treated PD animals following the administration of MK-801 (A and B).Values are means ± SD (n = 5, *P<0.02; **P<0.002). Scale bar: 20 µm.

NPCs in the SVZ were immunostained with BrdU to investigate the effect of levodopa treatment on neurogenesis in vivo. The immunohistochemical analysis revealed a significant decrease in BrdU-positive cells in MPTP-treated mice compared to the control animals; however, BrdU-positive cells were elevated in the MPTP-mice treated with levodopa or PPX compared to the MPTP-only mice (Fig. 7A). Most BrdU-positive cells in the SVZ were co-localized with Ki-67, a proliferation marker in each animal group (Fig. S4). Stereological analysis revealed that decreased number of BrdU-positive cells in the SVZ of MPTP-treated mice relative to the controls was more evident, and the number of BrdU positive cells was much greater in the PPX-treated mice than in the levodopa-treated mice (Fig. 7B). Furthermore, levodopa treatment significantly decreased the number of BrdU-positive cells in in MPTP untreated mice compared to the control group (Fig. S5A), whereas co-administration of MK-801 and L-dopa did not change neurogenic activity in MPTP untreated mice (Fig. S5B).

Additionally, we analyzed whether MK-801 would lead to modulate the decrease in levodopa treatment-associated neurogenesis. We found that MK-801 administration significantly increased the number of BrdU-positive cells in the SVZ of levodopa-treated PD mice (Fig. 7A and B). Meanwhile, MK-801 administration did not significantly change neurogenic activity in the PPX-treated PD animals (Fig. 7A and B) or MPTP-only treated mice (Fig. S6).

## Discussion

This is the first study to evaluate the effect of levodopa-induced hyperhomocysteinemia on neurogenesis in vitro and in vivo The major findings were (1) elevated homocysteine by levodopa treatment exerted an anti-proliferative effect on NPCs in the SVZ, (2) levodopa-induced apoptosis in the NPCs is mediated by ERK signaling pathways possibly via the NMDA receptor, and (3) the dopamine agonist produced a greater increase in neurogenesis compare to levodopa.

Accumulating clinical evidence suggests that chronic administration of levodopa in patients with PD lead to increase the homocysteine synthesis. Our data showing that levodopa treatment stimulated the release of homocysteine from cultured astrocytes and increased homocysteine concentration in the plasma and brain of MPTP-treated PD animals are consistent with those findings. Unexpectedly, the level of brain homocysteine in this study was significantly decreased in MPTP-treated mice compared to controls. This may be ascribed to decreased basal metabolism in catechol-O-methyltransferase (COMT)-mediated of endogenous dopamine due to dopamine depletion and alterations in methylation index by MPTP treatment [27]–[29]. Experimental studies indicate that homocysteine acts as an excitatory amino-acid by activating NMDA receptors [30]. Thus, homocysteine induces mitochondrial dysfunction, free radicals and cytosolic calcium accumulation, and activation of apoptotic pathways [13], [31], [32]. Accordingly, preclinical evidence has suggested that hyperhomocysteinemia associated with levodopa treatment has a detrimental effect on both dopaminergic and non-dopaminergic neurons in PD models [33]. However, whether levodopa-induced hyperhomocysteinemia may contribute to accelerate the progression of nigral motor dysfunction and the risk of extra nigral non-motor features in patients with PD is controversial and remains to be determined. In this regard, scientific evidence addressing the metabolic consequences of levodopa treatment on other non-dopaminergic systems, such as neurogenic system is critical for the development of sophisticated therapeutic strategies for PD.

Our in vitro data demonstrated that the increase in homocysteine release in levodopa-treated astrocytes had a neurotoxic effect on NPCs in the SVZ. Furthermore, the homocysteine-induced phosphorylation of ERK via the NMDA receptors led to NPC apoptosis. In accordance with previous studies [34], [35], the NPCs isolated from the SVZ in our study expressed NMDA receptor 2A-C subunits as well as the NR1 subunit. The role of the NMDA receptor in regulating an upstream MAPK superfamily and ERK-mediated proapoptotic signals has been extensively investigated. In NMDA receptor mediated neuronal toxicity, largely via the NMDAR-mediated influx of extracellular Ca2+, ERK-MAP kinase is known to be rapidly and transiently activated, and be involved in glutamate-induced apoptosis [36], [37]. Furthermore, Poddar and Paul [14] demonstrated recently that elevated homocysteine would lead to NMDA receptor-mediated neuronal cell death through sustained activation of ERK-MAP kinase. Our in vitro study supported these findings by showing that treatment with a NMDA antagonist (MK-801) significantly attenuated levodopa-induced activation of ERK signaling pathways and apoptotic cell death in the NPCs. Accordingly, these data suggest that elevated homocysteine by levodopa treatment has an important role in antiproliferative effect on NPCs possibly through NMDA receptor mediated the activation of ERK-dependent proapoptotic pathways.

To evaluate the NMDA receptor modulation on SVZ neurogenesis, we extended our study to an animal model of PD using MPTP. As expected, neurogenesis in the SVZ as measured by BrdU-positive cells was significantly reduced in the MPTP-treated PD animals. This finding agrees with previous studies reporting a relationship between dopamine depletion and decreased neurogenesis in the SVZ in the postmortem brains of patients with PD and animal models of PD [3], [4]. Interestingly, our in vivo data demonstrated that treatment with a NMDA antagonist significantly increased neurogenesis in the SVZ of levodopa-treated PD mice compared to that in levodopa-only PD animals. Several lines of evidence have suggested that prolonged NMDA receptor activation might decrease the rate of proliferation and the number of newly generated neurons in the SVZ or dentate gyrus [38], [39]. However, the role of the NMDA receptor in regulating neurogenesis is inconsistent and likely depends on the NMDA receptor subtypes, aging, and exposure time or dose of NMDA agonists/antagonists [2], [40], [41]. Thus, the NMDA antagonist may attenuate the prolonged NMDA receptor activation produced by the levodopa-induced increase in homocysteine in the brain, and in turn lead to decreased apoptosis of NPCs in the SVZ. Our in vivo results further supports that elevated homocysteine by levodopa treatment would modulate NMDA-dependent neurogenesis. However, contradictory results have been reported for the NMDA subtypes underlying homocysteine-induced activation of ERK2 and regulation of neurogenesis; the former is mediated through NMDA 2A subunit and the latter is mediated via NMDA 2B subunit [14], [38]. Further study is needed to clarify the signaling pathways underlying homocysteine-mediated NMDA receptor dependent regulation of neurogenesis.

Another interesting finding is comparative analysis of neurogenic activity between levodopa and dopamine agonists. Recent studies have found that NPCs in the SVZ exhibited dopaminergic receptors and dopaminergic innervation, suggesting that they are important for the proliferation of NPCs in the SVZ. Importantly, the neurogenic effects are mediated by activation of the D3 dopamine receptors, whereby dopamine receptor activation induces ciliary neurotrophic factor release into neurogenic niches [42]. Adding to evidence that dopamine agonists augment neurogenesis in animal models of PD [43], O’Sullivan et al. [44] very recently demonstrated a positive impact of chronic levodopa use on the number of neural stem cells in the SVZ of patients with PD. To date, however, there are no available studies dealing with a direct comparison of neurogenic activity between levodopa and dopamine agonist in the same experimental design. In the present study, we found that the number of cultured NPCs and BrdU-positive cells in the SVZ of the PD animals was significantly higher in PPX treatment group than in levodopa treatment group, indicating that the neurogenic activity of the dopamine agonist was superior to that of levodopa. The difference in neurogenic activity may be the result of the detrimental effect of homocysteine on NPCs by levodopa treatment. Moreover, as a member of the D2 receptor family, PPX has a higher affinity for the D3 dopamine receptor than dopamine [45], and this property may also contribute to difference in neurogenic activities, being in favor of dopamine agonist. Indeed, the number of BrdU-positive cells in the SVZ was higher in the PD animals treated with levodopa than in the PD mice that did not receive levodopa treatment. This finding may suggest that the pro-neurogenic effect of dopaminergic receptor stimulation by levodopa is stronger than the anti-neurogenic effect resulted from levodopa-induced hyperhomocysteinemia; however, further clinical evidence of postmortem study regarding the role of dopamine agonist in modulation of neurogenesis may clarify this issue.

Regarding the functional impact of adult neurogenesis, several lines of evidence have suggested that adult neurogenesis plays a regulatory role in olfaction, mood, and memory. In the PD animal model, Van Kampen and Eckman [6] reported that an increase in neurogenesis following treatment with a dopaminergic drug restored the nigrostriatal dopaminergic projection concomitant with function motor recovery, even though a debate exists as to whether NPCs from the SVZ would transdifferentiate into nigral dopaminergic phenotypes or migrate into the striatum and then incorporate into host neurons [46]. From a therapeutic perspective, the modulation of endogenous adult neurogenesis to repair the damaged PD brain would have a significant impact in future strategy of PD treatment. Furthermore, the neurogenic activity of the dopaminergic drugs commonly used to treat patients with PD must be a consideration for clinical practice along with neuroprotection, dopaminergic medication-associated motor complications, and dyskinesia. In this regard, our finding that dopamine agonists have greater neurogenic activity than levodopa is clinically relevant for human PD treatment. Future studies are needed to determine whether inhibition of homocysteine synthesis using clinically available COMT inhibitor can protect NPCs against levodopa-induced hyperhomocysteinemia.

In summary, the present study demonstrated that a levodopa-induced increased in homocysteine has a detrimental effect on adult neurogenesis via NMDA receptor-mediated ERK signaling pathways, and modulation of levodopa-induced hyperhomocysteinemia by a NMDA antagonist or dopaminergic agonist is clinically relevant for human PD treatment in terms of adult neurogenesis. Future studies focusing on the modulation of adult neurogenesis using small molecule receptor agonists would extend the clinical applications of our findings.

## Supporting Information

Figure S1 **In vivo study design.** At 6 weeks of age, the mice received subchronic injections of MPTP freshly dissolved in normal saline (25 mg/kg/day as the free base in normal saline by intraperitoneal injection (i.p). On day 28 after the first administration of levodopa, PPX, or normal saline, all animals were assigned to a BrdU injection. BrdU was administered i.p once daily on five subsequent days.
(DOC)

Figure S2 **The viability of astrocytes and NPCs.** The astrocytes were treated with different doses of levodopa (A), pramipexol (B), and MK-801 (C) to identify a dose that did not induce cell death. Values are means ± SD (n = 3/group, *P<0.005).
(DOC)

Figure S3 **The direct effects of homocysteine(Hcy) on the NPCs.** The NPCs were treated with varying doses of homocysteine in 72 hrs to determine the direct effects of homocysteine on the viability of NPCs. Homocysteine treatment decreased the viability of NPCs in a dose-dependent manner (A). Western blot analysis revealed that homocysteine treatment increased the expression of phosphorylated ERK in a dose-dependent manner (B) and the expression of phosphorylated ERK was decreased in presence NMDA antagonist, MK-801 (C). The treatment of SCH-23390, a D1 receptor antagonist, did not decrease the expression of phosphorylated ERK (D), which suggest that ERK activation is not mediated via D1 receptor. Values are means ± SD (n = 3/group, *P<0.05, **P<0.01).
(DOC)

Figure S4 **Double immunochemical analysis of BrdU antibody and Ki-67 antibody in each animal group**. The immunohistochemical analysis revealed that most BrdU-positive cells in the subventricular zone (SVZ) were co-localized with Ki-67 in control, MPTP-only treated, MPTP and levodopa treated, MPTP and PPX treated groups. The number of Ki-67-positive cells in the SVZ in each animal group did not differ significantly compared with BrdU-positive cells.
(DOC)

Figure S5 **The effects of levodopa treatment on the level of homocysteine and neurogenesis in MPTP untreated mice.** Levodopa treatment or co-administration of MK-801 and L-dopa in MPTP untreated mice led to increase in both plasma and brain homocysteine levels compared to the control (A). Immunohistochemical analysis revealed that levodopa treatment significantly decreased the number of BrdU-positive cells in in MPTP untreated mice compared to the control group, whereas co-administration of MK-801 and levodopa did not change neurogenetic activity in MPTP untreated mice compared to controls (B). And Values are means ± SE (n = 4; *P<0.05, **P<0.01).
(DOC)

Figure S6 **The modulatory effect of NMDA activation solely induced by MTPT treatment on neurogenic activity.** Immunohistochemical (A) and stereological (B) analyses revealed that the number of BrdU-positive cells in MPTP-only treated mice not differ significantly compared with those in MPTP and MK-801-treated mice. And Values are means ± SE (n = 4; **P<0.01).
(DOC)
