# Supplementary material for: Elevated Homocysteine by Levodopa Is Detrimental to Neurogenesis in Parkinsonian Model
Source: PLoS One. 2012 Nov 28;7(11):e50496. doi: 10.1371/journal.pone.0050496 (PMC3509089; doi:10.1371/journal.pone.0050496)
Supplement: Figure S1 — In vivo study design. At 6 weeks of age, the mice received subchronic injections of MPTP freshly dissolved in normal saline (25 mg/kg/day as the free base in normal saline by intraperitoneal injection (i.p). On day 28 after the first administration of levodopa, PPX, or normal saline, all animals were assigned to a BrdU injection. BrdU was administered i.p once daily on five subsequent days. (DOC) [file pone.0050496.s001.doc]

***Figure S1***

**
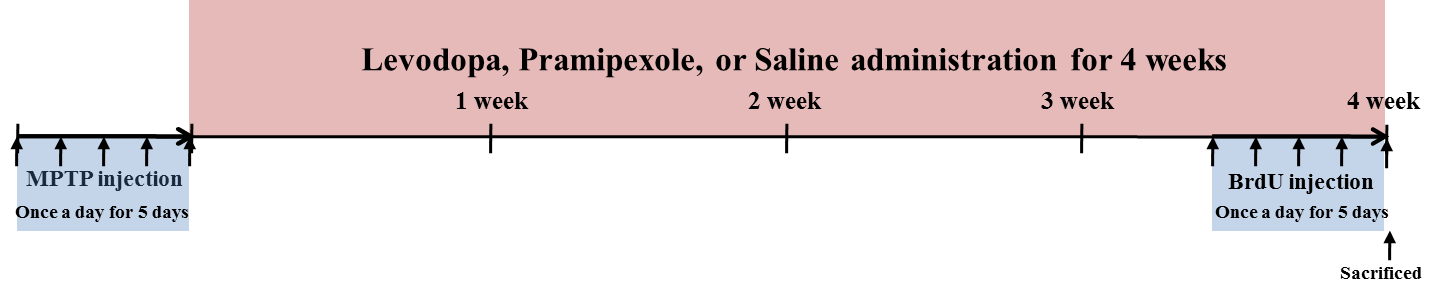
**

**Figure S1. In vivo study design.** At 6 weeks of age, the mice received subchronic injections of MPTP freshly dissolved in normal saline (25 mg/kg/day as the free base in normal saline by intraperitoneal injection (i.p). On day 28 after the first administration of levodopa, PPX, or normal saline, all animals were assigned to a BrdU injection. BrdU was administered i.p once daily on five subsequent days.
