# Supplementary material for: Elevated Homocysteine by Levodopa Is Detrimental to Neurogenesis in Parkinsonian Model
Source: PLoS One. 2012 Nov 28;7(11):e50496. doi: 10.1371/journal.pone.0050496 (PMC3509089; doi:10.1371/journal.pone.0050496)
Supplement: Figure S2 — The viability of astrocytes and NPCs. The astrocytes were treated with different doses of levodopa (A), pramipexol (B), and MK-801 (C) to identify a dose that did not induce cell death. Values are means ± SD (n = 3/group, *P<0.005). (DOC) [file pone.0050496.s002.doc]

***Figure S2***


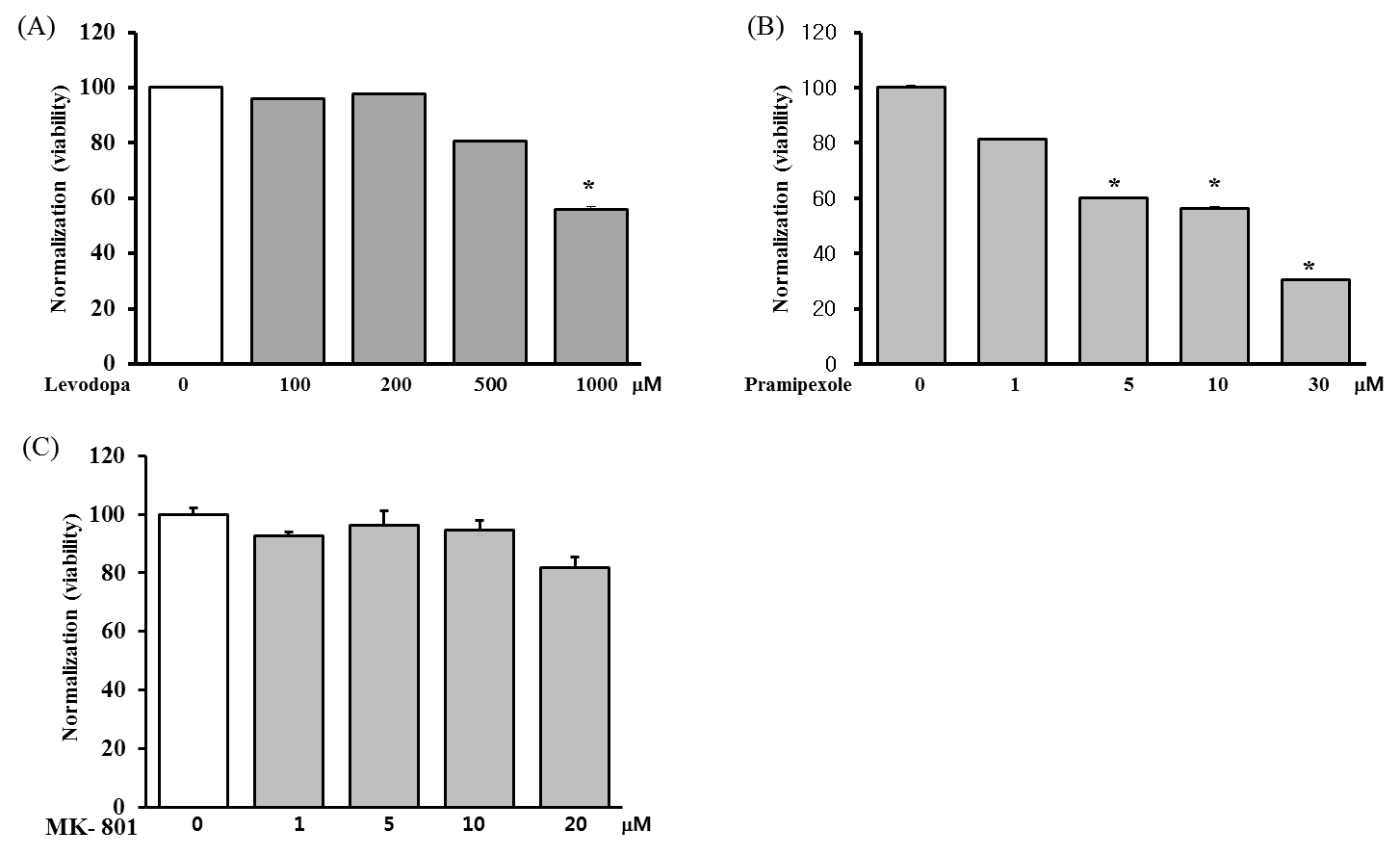


**Figure S2. The viability of astrocytes and NPCs.** The astrocytes were treated with different doses of levodopa (A), pramipexol (B), and MK-801 (C) to identify a dose that did not induce cell death. Values are means ± SD (n=3/group, *P < 0.005).
