# Supplementary material for: Elevated Homocysteine by Levodopa Is Detrimental to Neurogenesis in Parkinsonian Model
Source: PLoS One. 2012 Nov 28;7(11):e50496. doi: 10.1371/journal.pone.0050496 (PMC3509089; doi:10.1371/journal.pone.0050496)
Supplement: Figure S3 — The direct effects of homocysteine(Hcy) on the NPCs. The NPCs were treated with varying doses of homocysteine in 72 hrs to determine the direct effects of homocysteine on the viability of NPCs. Homocysteine treatment decreased the viability of NPCs in a dose-dependent manner (A). Western blot analysis revealed that homocysteine treatment increased the expression of phosphorylated ERK in a dose-dependent manner (B) and the expression of phosphorylated ERK was decreased in presence NMDA antagonist, MK-801 (C). The treatment of SCH-23390, a D1 receptor antagonist, did not decrease the expression of phosphorylated ERK (D), which suggest that ERK activation is not mediated via D1 receptor. Values are means ± SD (n = 3/group, *P<0.05, **P<0.01). (DOC) [file pone.0050496.s003.doc]

***Figure S3***

**
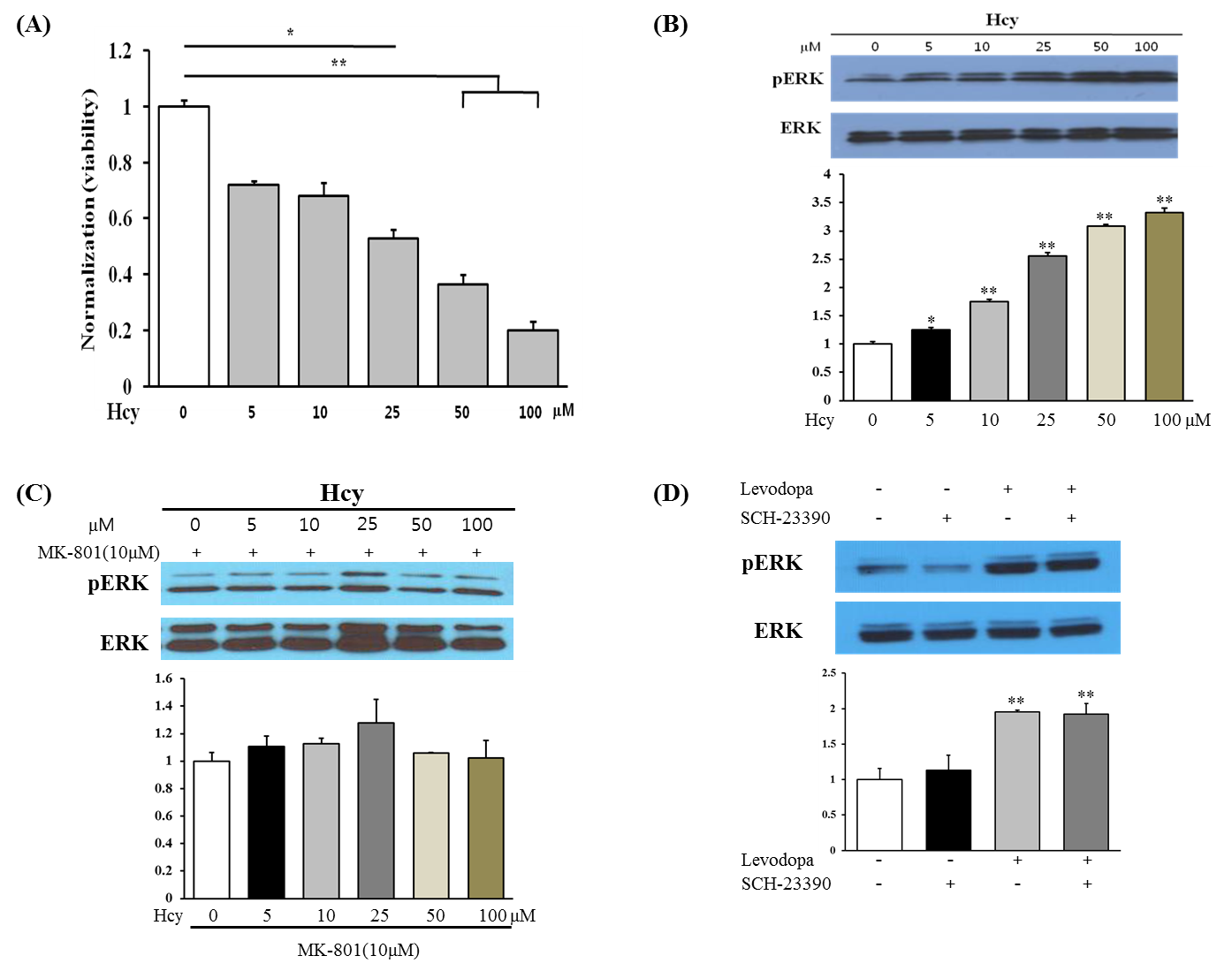
**

**Figure S3. The direct effects of homocysteine(Hcy) on the NPCs.** The NPCs were treated with varying doses of homocysteine in 72hrs to determine the direct effects of homocysteine on the viability of NPCs. Homocysteine treatment decreased the viability of NPCs in a dose-dependent manner (A). Western blot analysis revealed that homocysteine treatment increased the expression of phosphorylated ERK in a dose-dependent manner (B) and the expression of phosphorylated ERK was decreased in presence NMDA antagonist, MK-801 (C). The treatment of SCH-23390, a D1 receptor antagonist, did not decrease the expression of phosphorylated ERK (D), which suggest that ERK activation is not mediated via D1 receptor. Values are means ± SD (n=3/group, *P <0.05, **P < 0.01).
