# Supplementary material for: Elevated Homocysteine by Levodopa Is Detrimental to Neurogenesis in Parkinsonian Model
Source: PLoS One. 2012 Nov 28;7(11):e50496. doi: 10.1371/journal.pone.0050496 (PMC3509089; doi:10.1371/journal.pone.0050496)
Supplement: Figure S4 — Double immunochemical analysis of BrdU antibody and Ki-67 antibody in each animal group. The immunohistochemical analysis revealed that most BrdU-positive cells in the subventricular zone (SVZ) were co-localized with Ki-67 in control, MPTP-only treated, MPTP and levodopa treated, MPTP and PPX treated groups. The number of Ki-67-positive cells in the SVZ in each animal group did not differ significantly compared with BrdU-positive cells. (DOC) [file pone.0050496.s004.doc]

***Figure S4***

**
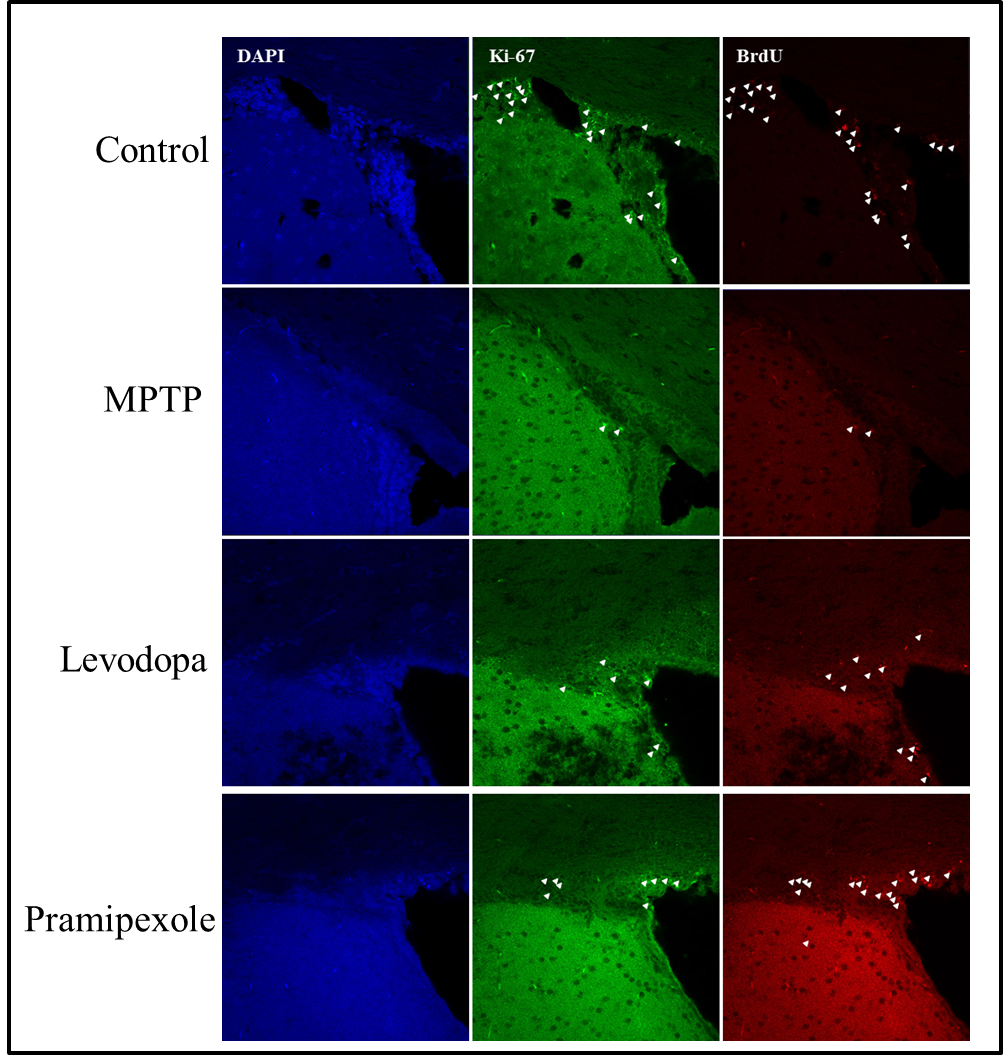
**

**Figure S4. Double immunochemical analysis of BrdU antibody and Ki-67 antibody in each animal group**. The immunohistochemical analysis revealed that most BrdU-positive cells in the subventricular zone (SVZ) were co-localized with Ki-67 in control, MPTP-only treated, MPTP and levodopa treated, MPTP and PPX treated groups. The number of Ki-67-positive cells in the SVZ in each animal group did not differ significantly compared with BrdU-positive cells.
