# Supplementary material for: Elevated Homocysteine by Levodopa Is Detrimental to Neurogenesis in Parkinsonian Model
Source: PLoS One. 2012 Nov 28;7(11):e50496. doi: 10.1371/journal.pone.0050496 (PMC3509089; doi:10.1371/journal.pone.0050496)
Supplement: Figure S5 — The effects of levodopa treatment on the level of homocysteine and neurogenesis in MPTP untreated mice. Levodopa treatment or co-administration of MK-801 and L-dopa in MPTP untreated mice led to increase in both plasma and brain homocysteine levels compared to the control (A). Immunohistochemical analysis revealed that levodopa treatment significantly decreased the number of BrdU-positive cells in in MPTP untreated mice compared to the control group, whereas co-administration of MK-801 and levodopa did not change neurogenetic activity in MPTP untreated mice compared to controls (B). And Values are means ± SE (n = 4; *P<0.05, **P<0.01). (DOC) [file pone.0050496.s005.doc]

***Figure S5***

**
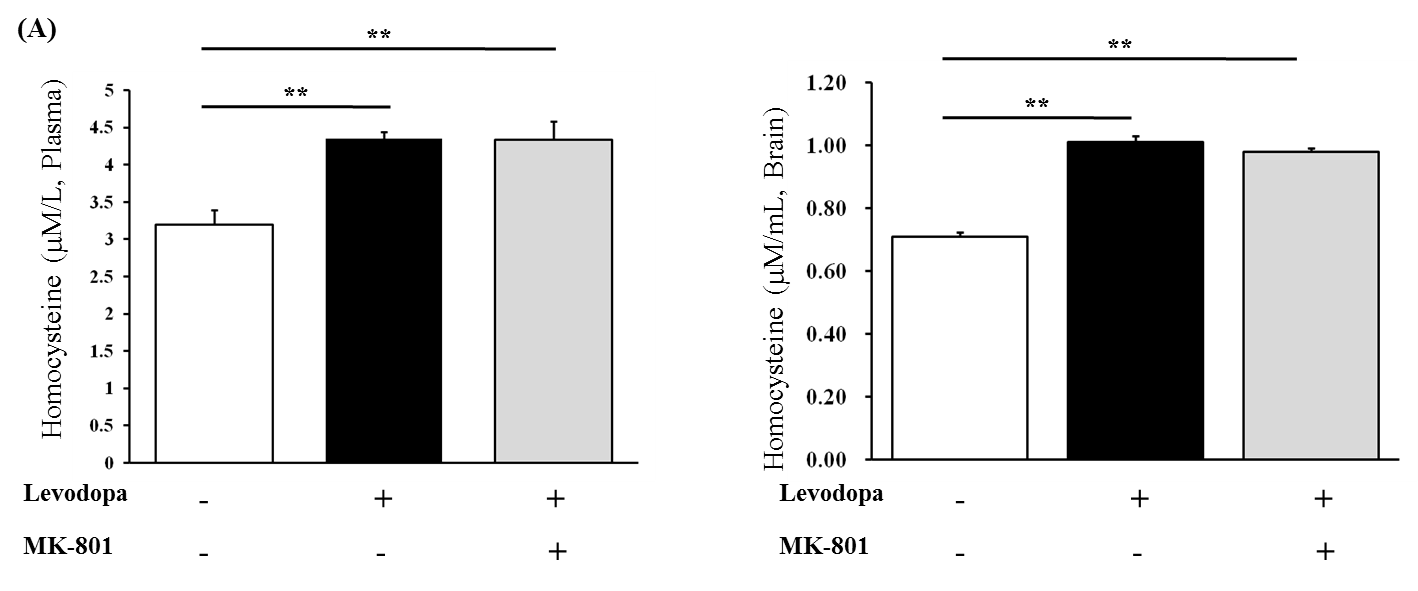

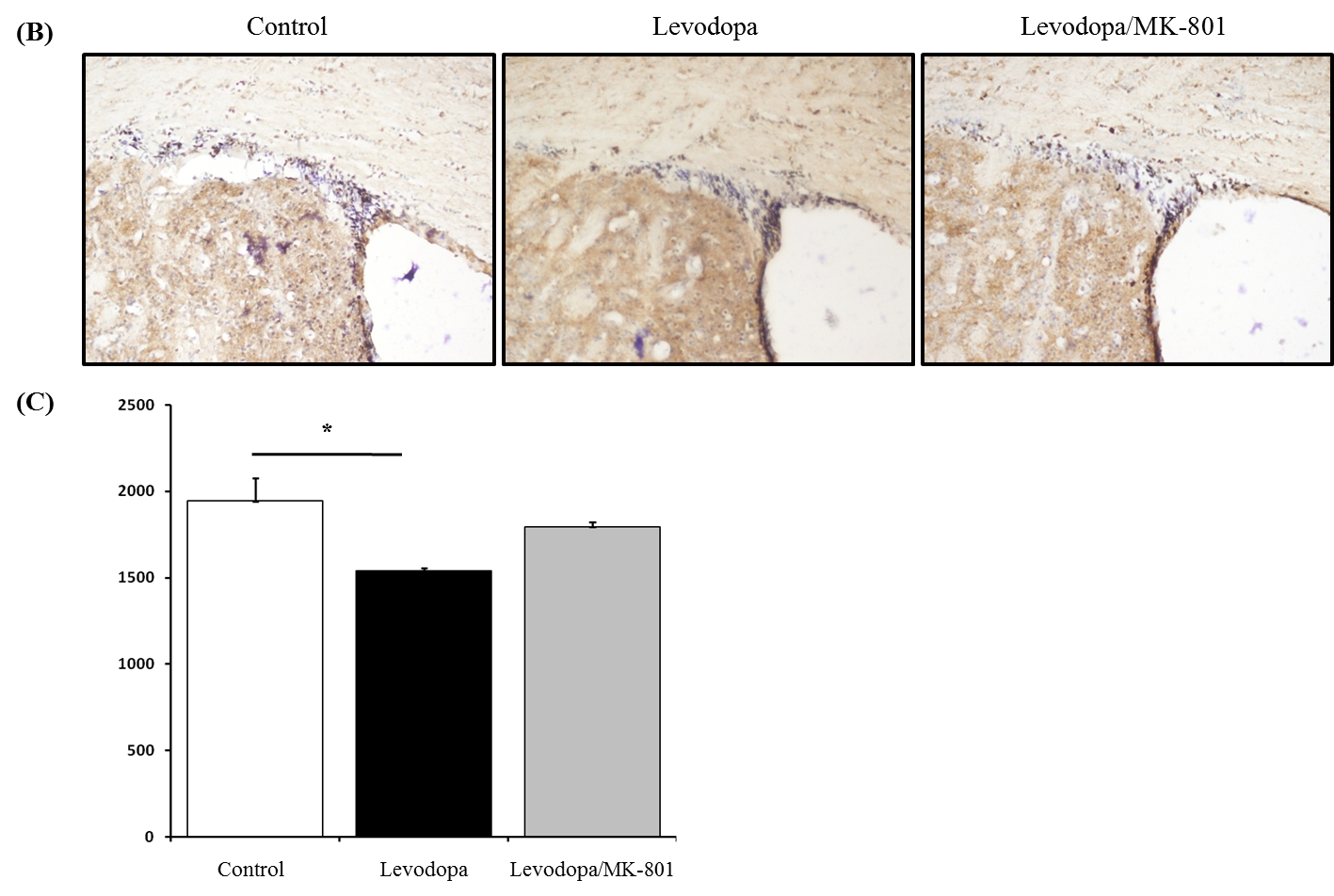
**

**Figure S5. The effects of levodopa treatment on the level of homocysteine and neurogenesis in MPTP untreated mice.** Levodopa treatment or co-administration of MK-801 and L-dopa in MPTP untreated mice led to increase in both plasma and brain homocysteine levels compared to the control (A). Immunohistochemical analysis revealed that levodopa treatment significantly decreased the number of BrdU-positive cells in in MPTP untreated mice compared to the control group, whereas co-administration of MK-801 and levodopa did not change neurogenetic activity in MPTP untreated mice compared to controls (B). And Values are means ± SE (n=4; *P <0.05, **P < 0.01).
