# Supplementary material for: Elevated Homocysteine by Levodopa Is Detrimental to Neurogenesis in Parkinsonian Model
Source: PLoS One. 2012 Nov 28;7(11):e50496. doi: 10.1371/journal.pone.0050496 (PMC3509089; doi:10.1371/journal.pone.0050496)
Supplement: Figure S6 — The modulatory effect of NMDA activation solely induced by MTPT treatment on neurogenic activity. Immunohistochemical (A) and stereological (B) analyses revealed that the number of BrdU-positive cells in MPTP-only treated mice not differ significantly compared with those in MPTP and MK-801-treated mice. And Values are means ± SE (n = 4; **P<0.01). (DOC) [file pone.0050496.s006.doc]

**Figure S6.**

**
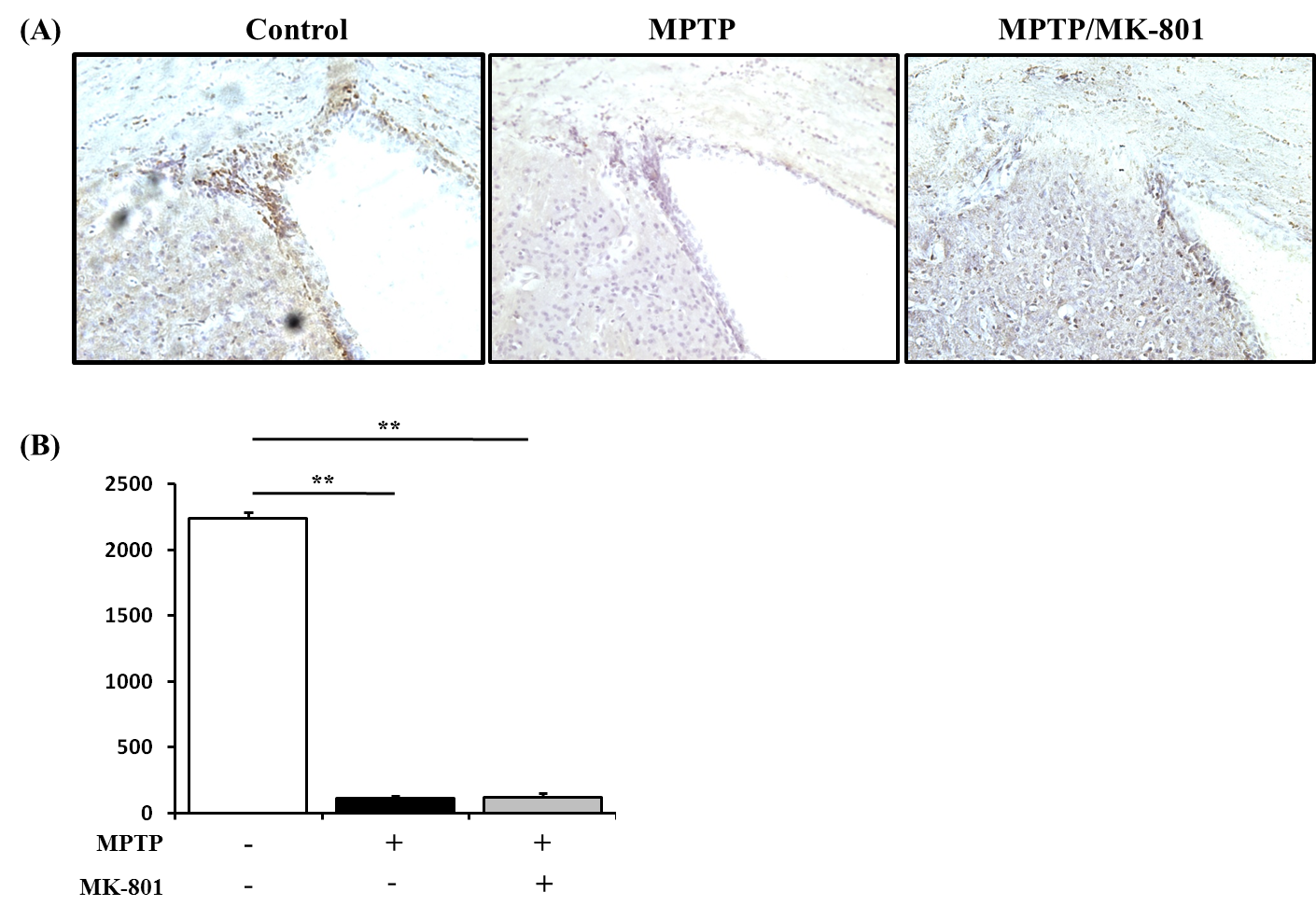
**

**Figure S6. The modulatory effect of NMDA activation solely induced by MTPT treatment on neurogenic activity.** Immunohistochemical (A) and stereological (B) analyses revealed that the number of BrdU-positive cells in MPTP-only treated mice not differ significantly compared with those in MPTP and MK-801-treated mice. And Values are means ± SE (n=4; **P < 0.01).
